# 

**Published:** 1906-02

**Authors:** 


### BOOKS RECEIVED.

International Clinics. A Quarterly of Illustrated Clinical Lectures and especially prepared articles on Treatment, Medicine, Surgery, Neurology, Pediatrics, Obstetrics, Gynecology, Orthopedics, Pathology, Dermatology, Opthamology, Otology, Rhinology, Laryngology, Hygiene and other topics of interest to students and practitioners. By leading members of the medical profession throughout the world. Edited by A. O. J. Kelly, A.M., M.D., Philadelphia. Volume IV. Fifteenth series. 1905. Philadelphia and London: J. B. Lippincott Co. (Cloth, $2.00).

On the Nature, Causes, Variety and Treatment of Bodily Deformities. By the late E. J. Chance. Edited by John Poland, F.R.C.S. A series of lectures. Second edition. In two volumes—Vol. I. Duodecimo, pp. 314. London: Smith, Elder & Co., 15 Waterloo Pl., S. W. 1905. (Price. 6| net. $1.50).

Thornton's Pocket Medical Formulary (heretofore known as The Medical News Pocket Formulary). New (7th) edition, revised to accord with the new U. S. Pharmacopeia, containing more than 2,000 prescriptions with indications for their use. In one leather bound volume. Lea, Brothers & Co., Publishers, Philadelphia and New York. 1906. (Price: $1.50 net).

Thirty-first Annual Report of the Secretary of the Michigan State Board of Health; for the fiscal year ending June 30, 1903. Lansing: Robert Smith Publishing Co. 1904.

Modern Clinical Medicine. Diseases of Metabolism and of the Blood. Animal Parasites. Toxicology. Edited by Richard C. Cabot, M.D., instructor in Clinical Medicine in the Medical School of Harvard University. Authorised translation from "Die Deutsche Klinik," under the general editorial supervision of Julius L. Salinger, M.D. With one colored plate and 58 illustrations in the text. Octavo, pp. 650. New York and London: D. Appleton & Co. 1906. (Price: Cloth, $6.00).

Koplik on Diseases of Children. A Treatise on the Diseases of Infancy and Childhood. For Students and Physicians. By Henry Koplik, M.D., Pediatrist to Mt. Sinai Hospital, ex-President American Pediatric Society, etc., New York. New (2d) edition. Revised and enlarged in text and illustrations. Octavo, 868 pages, 184 engravings and 33 plates. Lea, Brothers & Co., Publishers, Philadelphia and New York, 1906. (Cloth, $5.00; Leather, $6.00 net).

Diseases of Infancy and Childhood, by L. Emmett Holt, M.D., S.D., LL.D., Professor of Diseases of Children in the College of Physicians of Columbia University, New York. With 241 illustrations, including eight colored plates. This edition revised and enlarged. Octavo, pp. 1174. New York and London: D. Appleton. 1906.

A Laboratory Manual of Physiological Chemistry. By Elbert W. Rockwood, M.D., Ph.D., Professor of Chemistry and Toxicology and Head of the Department of Chemistry in the University of Iowa. Second Edition, Revised and Enlarged. With one colored plate and three plates of microscopic preparations. Large 12mo, 229 pages, Extra Cloth. F. A. Davis Company, Publishers, 1914 Cherry St., Philadelphia, Pa. (Price: $1.00 net).

The Practice of Medicine. A textbook for practitioners and students, with special reference to diagnosis and treatment. By James Tyson, M.D., Professor of Medicine in the University of Pennsylvania, and physician to the Hospital of the University; physician to the Pennsylvania Hospital, etc. Fourth Edition, revised and enlarged, with 240 illustrations, including colored plates. Octavo, pp. 1350. Philadelphia: P. Blakiston's Son & Company, 1012 Walnut St. 1906. (Price: Cloth, $5.50).

The Physical Examination of Infants and Young Children. By Theron Wendell Kilmer, M.D., Adjunct Attending Pediatrist to the Sydenham Hospital; Instructor in Pediatrics in the New York Polyclinic Medical School and Hospital, New York; Attending Physician to the Summer Home of St. Giles, Garden City, New York. Illustrated with 59 Half-tone Engravings. 12mo., 86 Pages. Bound in Extra Cloth. F. A. Davis Company, Publishers, 1914-16 Cherry St., Philadelphia, Pa. (Price: $ .75 net).

The Influence of the Menstrual Function on Certain Diseases of the Skin. By L. Duncan Bulkley, A.M., M.D., Physician to the New York Skin and Cancer Hospital, Consulting Physician to the New York Hospital, Consulting Dermatologist to the Randall's Island Hospitals, to the Manhattan Eye and Ear Hospital, and to the Hospital for Ruptured and Crippled, etc. Demi-octavo, pp. 108. New York and London, Rebman Company, 1123 Broadway. (Price: $1.00).

Christianity and Sex Problems. By Hugh Northcote, M.A. Crown Octavo, 257 pages. Bound in Extra Cloth. F. A. Davis Company, Publishers. 1914-16 Cherry St., Philadelphia, Pa. (Price $2.00 net).

On the Relation of Diseases of the Skin to Internal Disorders. ith observations on Diet, Hygiene and General Theraputics; forming a supplementary volume to every manual and textbook of Dermatologyn. By L. Duncan Bulkley, A.M., M.D., Physician to the New York Skin and Cancer Hospital, Consulting Physician to the New York Hospital, Consulting Dermatologist to the Randall's Island Hospitals, to the Manhattan Eye and Ear Hospital and to the Hospital for Ruptured and Crippled, etc. Demioctavo, pp. xv-175. New York and London: Rebman Company, 1123 Broadway. (Price: $1.50).

Nasal Sinus Surgery with Operations on Nose and Throat. By Beaman Douglas, M.D., Professor of Diseases of the Nose and Throat in the New York Post-Graduate Medical School and Hospital. Illustrated with 68 full-page Half-tone and Colored Plates, including nearly 100 Figures. Royal Octavo, 256 Pages. Bound in Extra Cloth. F. A. Davis Company, Publishers, 1914-16 Cherry St., Philadelphia, Pa. (Price: $2.50 net).

The Ophthalmoscope and How to Use It. By James Thorington, A.M., M.D., Professor of Diseases of the Eye in the Philadelphia Polyclinic; Ophthalmologist to the Elwyn, Vineland, and New Jersey State Training Schools for Feeble-minded Children, etc. Author of "Rrefraction and How to Refract," and "Retinoscopy." 12 Colored Plates and other illustrations. 8vo. pp. 398. Philadelphia: P. Blakiston's Son & Company. 1906. (Cloth, $2.50 net).

